# Concordance and discordance of sequence survey methods for molecular epidemiology

**DOI:** 10.7717/peerj.761

**Published:** 2015-02-17

**Authors:** Eduardo Castro-Nallar, Nur A. Hasan, Thomas A. Cebula, Rita R. Colwell, Richard A. Robison, W. Evan Johnson, Keith A. Crandall

**Affiliations:** 1 Computational Biology Institute, George Washington University, Ashburn, VA, USA; 2 CosmosID, College Park, MD, USA; 3 University of Maryland Institute for Advanced Computer Studies, University of Maryland, College Park, MD, USA; 4 Department of Biology, Johns Hopkins University, Baltimore, MD, USA; 5 Bloomberg School of Public Health, Johns Hopkins University, Baltimore, MD, USA; 6 Department of Microbiology and Molecular Biology, Brigham Young University, Provo, UT, USA; 7 Division of Computational Biomedicine, Boston University School of Medicine, Boston, MA, USA

**Keywords:** Phylogenomics, Data type, MLST, SNP, Phylogeography, Genomes, Molecular epidemiology, Bioterrorism, Biological weapons, High-throughput sequencing

## Abstract

The post-genomic era is characterized by the direct acquisition and analysis of genomic data with many applications, including the enhancement of the understanding of microbial epidemiology and pathology. However, there are a number of molecular approaches to survey pathogen diversity, and the impact of these different approaches on parameter estimation and inference are not entirely clear. We sequenced whole genomes of bacterial pathogens, *Burkholderia pseudomallei*, *Yersinia pestis*, and *Brucella spp*. (60 new genomes), and combined them with 55 genomes from GenBank to address how different molecular survey approaches (whole genomes, SNPs, and MLST) impact downstream inferences on molecular evolutionary parameters, evolutionary relationships, and trait character associations. We selected isolates for sequencing to represent temporal, geographic origin, and host range variability. We found that substitution rate estimates vary widely among approaches, and that SNP and genomic datasets yielded different but strongly supported phylogenies. MLST yielded poorly supported phylogenies, especially in our low diversity dataset, i.e., *Y. pestis*. Trait associations showed that *B. pseudomallei* and *Y. pestis* phylogenies are significantly associated with geography, irrespective of the molecular survey approach used, while *Brucella spp*. phylogeny appears to be strongly associated with geography and host origin. We contrast inferences made among monomorphic (clonal) and non-monomorphic bacteria, and between intra- and inter-specific datasets. We also discuss our results in light of underlying assumptions of different approaches.

## Introduction

Genomic data coupled with phylogenetic methods have enhanced the ability to track infectious disease epidemics through space and time (Baker, Hanage & Holt, 2010). For example, studies have tracked and characterized epidemics occurring at different geographic scales, across local, regional, global, and even historical scales; investigating multidrug-resistant *Staphylococcus aureus* in hospital settings (Kos et al., 2012; Köser et al., 2012), inferring continental origins of food pathogens (Goss et al., 2014), explaining seasonal influenza dynamics (Lemey et al., 2014), and ancient oral pathogens (Warinner et al., 2014), respectively. Such studies provide valuable information regarding migration rates, directionalities of spread, unique variants, genetic diversity, and drug resistance, as well as informing policy-makers about infection patterns associated with human activities (Bos et al., 2011; Morelli et al., 2010; Zhang et al., 2010). Accordingly, applications of analytical tools to large datasets are abundant in clinical pathology, bioforensics, biosurveillance, and molecular epidemiology (Reimer et al., 2011; Wilson, Allard & Brown, 2013).

Whole-genome sequencing (WGS) has become an affordable approach for such studies (Bertelli & Greub, 2013; Chen et al., 2013; Cornejo et al., 2013; Croucher et al., 2013; Pérez-Lago et al., 2013; Sheppard et al., 2013; Wielgoss et al., 2013). New technologies make it possible to compile datasets that were not even dreamed of twenty years ago (Chewapreecha et al., 2014; Marttinen et al., 2012; Nasser et al., 2014; Sheppard et al., 2013) which, in turn, is prompting scientists to ask new questions regarding pathogen distribution, diversity, identification, origin, and phenotype (Butler et al., 2013; Castillo-Ramirez et al., 2012; Grad & Waldor, 2013; Holt et al., 2012; Hong et al., 2014; Spoor et al., 2013).

Because there are now a variety of molecular survey approaches (whole genome sequencing (WGS), multi-locus sequence typing (MLST), and single nucleotide polymorphism (SNP) data) with different costs and resolution abilities, we explored the impact of these different approaches on inferences of population dynamics, transmission patterns, and parameter estimation. For instance, tracking the origin of bioterrorism agents depends on identifying diagnostic mutations, as in the anthrax attacks of 2001 (Read et al., 2002), or accurately identifying the subspecies of origin (Hong et al., 2014), and understanding the extent to which sampling strategy and choice of molecular survey approach affects temporal and spatial inferences.

Here, we set out to investigate how molecular survey approaches compare, using three select agents as models, namely *Yersinia pestis* (causative agent of plague), *Burkholderia pseudomallei* (causative agent of melioidosis), and *Brucella* spp. (febrile disease). These bacterial species are relevant from health and biosecurity perspectives, and there exists a sizable amount of genomic and supporting information (date of collection, geographic location, and host) for them. Also, they allow for interesting contrasts including comparing intraspecific datasets (*Y. pestis* v. *B. pseudomallei*), one from monomorphic bacteria (clonal), and the other from polymorphic bacteria, as well as interspecific comparisons (*Y. pestis* and *B. pseudomallei* vs. *Brucella* spp.)

Thus, we present and analyze new draft genomic sequences for 20 *Brucella* spp., 20 *Y. pestis*, and 20 *B. pseudomallei* isolates, which we combine with publicly available genomes (totaling 115 genomes) to compare inferences on evolutionary relationships, dates and rates, and geographic and host structure. Do molecular survey approaches, as currently practiced, produce incongruent inferences? We performed a comparison with real-world examples using species that represent genetic diversities of relevance to clinical molecular epidemiology. We applied different molecular survey approaches (WGS, SNPs, MLST) to evaluate whether these can recover equivalent evolutionary relationships, evolutionary rates and divergence dates, and whether phylogenies inferred with these approaches represent equivalent geographic and host structures.

## Methods

### Strain selection and sequencing

DNA was isolated from 20 strains of *Burkholderia pseudomallei, Yersinia pestis*, and *Brucella* spp. from the Brigham Young University Select Agent Archive. Samples were selected for sequencing to provide a range of (1) time of isolation, (2) geographic spread, and (3) host association (Table 1). DNA isolation followed standard protocols for select agents and was conducted at the Brigham Young University BSL-3 facility. All DNA preparations received a Certification of Sterility (10% of the final DNA preparation from each isolate was plated for sterility on appropriate agar, and after a minimum of five days of incubation at 37 °C, the samples showed no growth, indicating they contained no viable organisms) before being prepared for sequencing.

**Table 1:** Summary of genomes sequenced and collected in this study. Metadata on strain source, host, location and date of collection also provided when available.

NCBI Accession number	Species	Strain	Source	Host	Location	Date of collection
SRX286342	*Burkholderia pseudomallei*	5	Public Health LaboratoryService, London	Sheep	Australia	1949
SRX286347	*Burkholderia pseudomallei*	6	Public Health LaboratoryService, London	Human	Bangladesh	1960
SRX286346	*Burkholderia pseudomallei*	9	Public Health LaboratoryService, London	Human	Pakistan	1988
SRX286345	*Burkholderia pseudomallei*	18	Public Health LaboratoryService, London	Monkey	Indonesia	1990
SRX286357	*Burkholderia pseudomallei*	24	Public Health LaboratoryService, London	Horse	France	1976
SRX286354	*Burkholderia pseudomallei*	25	Public Health LaboratoryService, London	Soil	Madagascar	1977
SRX286353	*Burkholderia pseudomallei*	31	Public Health LaboratoryService, London	Water drain	Kenya	1992
SRX286352	*Burkholderia pseudomallei*	33	Public Health LaboratoryService, London	Manure	France	1976
SRX286350	*Burkholderia pseudomallei*	35	Public Health LaboratoryService, London	Human	Vietnam	1963
SRX286348	*Burkholderia pseudomallei*	68	Public Health LaboratoryService, London	Human	Fiji	1992
SRX286359	*Burkholderia pseudomallei*	91	Public Health LaboratoryService, London	Sheep	Australia	1984
SRX286361	*Burkholderia pseudomallei*	104	Public Health LaboratoryService, London	Goat	Australia	1990
SRX286363	*Burkholderia pseudomallei*	208	Public Health LaboratoryService, London	Human	Ecuador	1990
SRX286364	*Burkholderia pseudomallei*	4075	Public Health LaboratoryService, London	Human	Holland	1999
SRX286418	*Burkholderia pseudomallei*	Darwin-035	Royal Darwin Hospital	Human	Australia	2003
SRX286420	*Burkholderia pseudomallei*	Darwin-051	Royal Darwin Hospital	Dog	Australia	1992
SRX286421	*Burkholderia pseudomallei*	Darwin-060	Royal Darwin Hospital	Pig	Australia	1992
SRX286422	*Burkholderia pseudomallei*	Darwin-077	Royal Darwin Hospital	Bird	Australia	1994
SRX286423	*Burkholderia pseudomallei*	Darwin-150	Royal Darwin Hospital	Soil	Australia	2006
SRX286344	*Burkholderia pseudomallei*	80800117	Utah Department of Health	Human	USA	2008
NC_017832.1 NC_017831.1	*Burkholderia pseudomallei*	1026b	hhayden@u.washington.edu	Human	Thailand	1993
NC_009078.1 NC_009076.1	*Burkholderia pseudomallei*	1106a	J. Craig Venter Institute (JCVI)	Human	Thailand	1993
NC_012695.1	*Burkholderia pseudomallei*	MSHR346	Joint Genome Institute/LANL Center	Human	Australia	1996
NC_006351.1 NC_006350.1	*Burkholderia pseudomallei*	k96243	Sanger Institute	Human	Thailand	1996
NC_018529.1 NC_018527.1	*Burkholderia pseudomallei*	BPC006	Third Military MedicalUniversity	Human	China	2008
NZ_CM000774.1 NZ_CM000775.1	*Burkholderia pseudomallei*	1106b	J. Craig Venter Institute (JCVI)	Human	Thailand	1996
NZ_CM000833.1 NZ_CM000832.1	*Burkholderia pseudomallei*	1710a	J. Craig Venter Institute (JCVI)	Human	Thailand	1996
NC_007435.1 NC_007434.1	*Burkholderia pseudomallei*	1710b	J. Craig Venter Institute (JCVI)	Human	Thailand	1999
NC_009074.1 NC_009075.1	*Burkholderia pseudomallei*	668	J. Craig Venter Institute (JCVI)	Human	Australia	1995
NZ_CM001156.1 NZ_CM001157.1	*Burkholderia pseudomallei*	Bp22	Genome Institute of Singapore	Human	Singapore	1989
NC_007651 NC_007650	*Burkholderia thailandensis*	E264	J. Craig Venter Institute (JCVI)	Soil	Thailand	1994
SRX278648	*Brucella abortus*	1004, Strain 2032	National Animal Disease Center	Bovine	Missouri, USA	1990
SRX278790	*Brucella abortus*	1007, Strain 2045	National Animal Disease Center	Bovine	Florida, USA	1990
SRX278791	*Brucella abortus*	1019, Strain 2038	National Animal Disease Center	Bovine	Tennessee, USA	1990
SRX278792	*Brucella abortus*	1022, Strain 2073	National Animal Disease Center	Bovine	Georgia, USA	1990
SRX278793	*Brucella abortus*	1146, Strain 8-953	National Animal Disease Center	Elk	Montana, USA	1992
SRX278794	*Brucella abortus*	1668, Strain 00-666	National Animal Disease Center	Elk	Wyoming, USA	2000
SRX278891	*Brucella abortus*	YELL-99-067	Idaho National Engineering and Environmental Laboratory	Bison (amniotic fluid)	Wyoming, USA	1999
SRX282032	*Brucella abortus*	1614, Strain Weinheimer 4	National Animal Disease Center	Bovine	Texas, USA	2000
SRX282039	*Brucella canis*	1107, Strain 1-107	National Animal Disease Center	Canine	Missouri, USA	1990
SRX282040	*Brucella melitensis*	1253, Strain Ether, L657	National Animal Disease Center	Caprine	Unknown	1994
SRX282041	*Brucella melitensis*	BA 4837	New Mexico Departmentof Health	Human	New Mexico, USA	2003
SRX282042	*Brucella melitensis*	70000565	Utah Department of Health	Blood, Human	Utah, USA	2000
SRX282044	*Brucella melitensis*	80600020	Utah Department of Health	Blood, Human	Utah, USA	2006
SRX282045	*Brucella melitensis*	80800076	Utah Department of Health	Human	California, USA	2008
SRX282046	*Brucella neotomae*	1156, Strain 5K33, ATCC#23459	National Animal Disease Center	Desert wood rat	Unknown, USA	1992
SRX282047	*Brucella ovis*	1117, Strain 1-507	National Animal Disease Center	Ovine	Georgia, USA	1991
SRX282048	*Brucella ovis*	1698, Strain 13551-2114; 1985:Dhyatt	National Animal Disease Center	Ovine (semen)	Ft. Collins, Colorado, USA	2001
SRX282050	*Brucella species*	70100304	Utah Department of Health	Blood, Human	USA- Utah	2001
SRX282053	*Brucella suis*	1103, Strain 2483	National Animal Disease Center	Porcine	South Carolina, USA	1990
SRX282057	*Brucella suis*	1108, Strain 1-138	National Animal Disease Center	Porcine	New Jersey, USA	1990
NC_016777.1 NC_016795.1	*Brucella abortus*	A13334	Macrogen	Bovine	Korea	Unknown
NC_006932.1 NC_006933.1	*Brucella abortus*	bv 1, 9-941	USDA	Bovine	Wyoming, USA	Unknown
NC_010740.1 NC_010742.1	*Brucella abortus*	S19	Crasta OR	Bovine	Unknown, USA	1923
NC_010103.1 NC_010104.1	*Brucella canis*	ATCC 23365	DOE Joint Genome Institute	Dog	Unknown	Unknown
NC_016796.1 NC_016778.1	*Brucella canis*	HSK A52141	National VeterinaryResearch and Quarantine	Dog	South Korea	Unknown
NC_012442.1 NC_012441.1	*Brucella melitensis*	ATCC 23457	Los Alamos National Lab	Human	India	1963
NC_017244.1 NC_017245.1	*Brucella melitensis*	M28	Chinese National Human GenomeCenter at Shanghai	Sheep	China	1955
NC_003317.1 NC_003318.1	*Brucella melitensis*	bv 1, 16M	Integrated Genomics Inc	Goat	Unknown, USA	Unknown
NC_007618.1 NC_007624.1	*Brucella melitensis*	bv. 1 Abortus 2308	Lawrence Livermore National Lab	Standard laboratory strain	Unknown	Unknown
NC_017246.1 NC_017247.1	*Brucella melitensis*	M5-90	Chinese National Human GenomeCenter at Shanghai	Standard laboratory strain	Unknown	Unknown
NC_017248.1 NC_017283.1	*Brucella melitensis*	bv. 3 NI	China Agricultural University	Bovine	Inner Mongolia, China	2007
CP001578.1 CP001579.1	*Brucella microti*	CCM 4915	Sudic S	Vole	Czech Republic	2000
NC_009505.1 NC_009504.1	*Brucella ovis*	ATCC 25840	J. Craig Venter Institute	Sheep	Australia	1960
NC_015858.1 NC_015857.1	*Brucella pinnipedialis*	B2/94	Zygmunt, M.S.	Seal	UK	1994
NC_016775.1 NC_016797.1	*Brucella suis*	VBI22	Harold R. Garner	Bovine, milk	Texas, USA	Unknown
NC_004311.2 NC_004310.3	*Brucella suis*	bv 1, 1330	J. Craig Venter Institute	Pig	Unknown, USA	1950
NC_010167.1 NC_010169.1	*Brucella suis*	ATCC 23445	Joint Genome Institute/LANL Center	Hare	UK	1951
NC_009667.1 NC_009668.1	*Ochrobactrum anthropi*	ATCC 49188	DOE Joint Genome Institute	Arsenical cattle-dipping fluid	Australia	1988
SRX282065	*Yersinia pestis*	4954	New Mexico Departmentof Health	Human	NM, USA	1987
SRX282089	*Yersinia pestis*	1901b	New Mexico Departmentof Health	Human	NM, USA	1983
SRX282090	*Yersinia pestis*	Java (D88)	Michigan State University	Unknown	Far East	Unknown
SRX282091	*Yersinia pestis*	Kimberley (D17)	Michigan State University	Unknown	Near East	Unknown
SRX282092	*Yersinia pestis*	KUMA (D11)	Michigan State University	Unknown	Manchuria, China	Unknown
SRX282093	*Yersinia pestis*	TS (D5)	Michigan State University	Unknown	Far East	Unknown
SRX282094	*Yersinia pestis*	8607116	New Mexico Departmentof Health	Dog	NM, USA	Unknown
SRX282095	*Yersinia pestis*	1866	New Mexico Departmentof Health	Squirrel	NM, USA	Unknown
SRX282096	*Yersina pestis*	4139	New Mexico Departmentof Health	Cat	NM, USA	1995
SRX286281	*Yersinia pestis*	4412	New Mexico Departmentof Health	Human	NM, USA	1991
SRX286283	*Yersinia pestis*	2965	New Mexico Departmentof Health	Human	NM, USA	1995
SRX286290	*Yersinia pestis*	2055	New Mexico Departmentof Health	Human	NM, USA	1998
SRX286302	*Yersinia pestis*	2106	New Mexico Departmentof Health	Human	NM, USA	2001
SRX286303	*Yersinia pestis*	2772	New Mexico Departmentof Health	Cat	NM, USA	1984
SRX286304	*Yersinia pestis*	3357	New Mexico Departmentof Health	Mountain lion	NM, USA	1999
SRX286305	*Yersinia pestis*	AS 2509	New Mexico Departmentof Health	Rodent	NM, USA	2004
SRX286306	*Yersinia pestis*	AS 200900596	New Mexico Departmentof Health	Rabbit, liver/spleen	United States, Santa Fe, NM	2009
SRX286307	*Yersinia pestis*	V-6486	New Mexico Departmentof Health	Llama	Las Vegas, NM, USA	Unknown
SRX286340	*Yersinia pestis*	KIM (D27)	Michigan State University	Human	Iran/Kurdistan	1968
SRX286341	*Yersinia pestis*	AS200901509	New Mexico Departmentof Health	Liver/spleen, prairie dog	Santa Fe, NM, USA	2009
NC_017168.1	*Yersinia pestis*	A1122	Los Alamos National Lab	Ground squirrel	California	1939
NC_010159.1	*Yersinia pestis*	Angola	J. Craig Venter Institute (JCVI)	Human	Angola	Unknown
NC_008150.1	*Yersinia pestis*	Antiqua	DOE Joint Genome Institute	Human	Congo	1965
PRJNA54473	*Yersinia pestis*	B42003004	J. Craig Venter Institute (JCVI)	Marmota baibacina	China	2003
PRJNA54563	*Yersinia pestis*	CA88-4125	DOE Joint Genome Institute	Human	California	1988
NC_003143.1	*Yersinia pestis*	CO92	Sanger Institute	Human/cat	Colorado	1992
NC_017154.1	*Yersinia pestis*	D106004	Chinese Center for DiseaseControl and Prevention	Apodemus chevrieri	Yulong County, China	2006
NC_017160.1	*Yersinia pestis*	D182038	Chinese Center for DiseaseControl and Prevention	Apodemus chevrieri	Yunnan, China	1982
PRJNA54471	*Yersinia pestis*	E1979001	J. Craig Venter Institute (JCVI)	Eothenomys miletus	China	1979
PRJNA54469	*Yersinia pestis*	F1991016	J. Craig Venter Institute (JCVI)	Flavus rattivecus	China	1991
PRJNA54399	*Yersinia pestis*	FV-1	The Translational GenomicsResearch Institute	Prairy dog	Arizona	2001
PRJNA55339	*Yersinia pestis*	India 195	DOE Joint Genome Institute	Human	India	Unknown
PRJNA54383	*Yersinia pestis*	IP275	The Institute for Genomic Research	Human	Madagascar	1995
NC_009708.1	*Yersinia pseudotuberculosis*	IP31758	J. Craig Venter Institute (JCVI)	Human	Russia	1966
PRJNA54475	*Yersinia pestis*	K1973002	J. Craig Venter Institute (JCVI)	Marmota himalaya	China	1973
PRJNA42495	*Yersinia pestis*	KIM D27	J. Craig Venter Institute (JCVI)	Human	Iran/Kurdistan	1968
NC_004088.1	*Yersinia pestis*	KIM10+	Genome Center of Wisconsin	Human	Iran/Kurdistan	1968
NC_017265.1	*Yersinia pestis*	Medievalis str. Harbin 35	Virginia Bioinformatics Institute	Human	China	1940
PRJNA54477	*Yersinia pestis*	MG05-1020	J. Craig Venter Institute (JCVI)	Human	Madagascar	2005
NC_005810.1	*Yersinia pestis*	Microtus 91001	Academy of Military Medical Sciences, The Institute of Microbiology andEpidemiology, China	Microtus brandti	China	1970
NC_008149.1	*Yersinia pestis*	Nepal516	Genome Center of Wisconsin	Human/soil	Nepal	1967
PRJNA55343	*Yersinia pestis*	Pestoides A	DOE Joint Genome Institute	Human	FSU	1960
PRJNA58619	*Yersinia pestis*	Pestoides F	DOE Joint Genome Institute	Human	FSU	Unknown
PRJNA55341	*Yersinia pestis*	PEXU2	Enteropathogen Resource Integration Center (ERIC) BRC	Rodent	Brazil	1966
PRJNA54479	*Yersinia pestis*	UG05-045	J. Craig Venter Institute (JCVI)	Human	Uganda	2005
PRJNA47317	*Yersinia pestis*	Z176003	CCDC	Marmota himalayana	Tibet	1976

The DNA samples were prepared for multiplexed (single-end, 82 cycles) sequencing using a Illumina GAIIx genome analyzer (Illumina Inc., San Diego, CA). For each isolate, genomic library preparations were generated using a Nextera DNA Sample Prep Kit. Post-library quality control and quantification was done using BioAnalyzer 2100 high-sensitivity chips and KAPA SYBR FAST Universal 2X qPCR Master Mix. Post processing of reads was performed by the RTA/SCS v1.9.35.0 and CASAVA 1.8.0. Reads were trimmed to the Q30 level using CLCBio’s quality_trim program, and CutAdapt v0.95 was used to excise adapter and transposon contamination.

All sequencing run data and metadata were deposited in the Sequence Read Archive (SRA) under three projects, SRP022877, SRP022862, and SRP023117 for *Y. pestis*, *Brucella* spp., and *B. pseudomallei*, respectively.

### Dataset collection

Short reads were quality filtered (average read quality >30 Phred) and mapped against reference genomes employing the Burrows–Wheeler Transform algorithm, as implemented in SOAP (Li et al., 2008). The resulting SAM/BAM files were filtered for duplicate reads that might have arisen by PCR, and consensus sequences were called in Geneious 6.1.6 (Kearse et al., 2012; Li et al., 2009). Additionally, we retrieved full genomes along with host, collection date, and country of origin metadata for *B. pseudomallei* (11), *Brucella spp.* (18) and *Y. pestis* (26) from GenBank, GOLD, IMG, Patric, Broad Institute, and Pathema databases and resources totaling 115 genomes (Table 1; geographic distribution in Map S1) (Benson et al., 2010; Brinkac et al., 2010; Gillespie et al., 2011; Liolios et al., 2008; Markowitz et al., 2012). From the assembled genomes we derived all datasets as described below.

Multi-locus sequence type markers for *B. pseudomallei*, namely ace, *gltB*, *gmhD*, *lepA*, *lipA*, *narK*, and *ndh* were retrieved from the PubMLST database (http://bpseudomallei.mlst.net). For *Brucella* spp., we resorted to markers used by Whatmore, Perrett & MacMillan (2007) i.e., *gap*, *aroA*, *glk*, *dnaK*, *gyrB*, *trpE*, *cobQ*, *omp25*, and *int-hyp*. Likewise, for *Y. pestis* we obtained markers from PubMLST (Yersinia spp.; http://pubmlst.org/yersinia/) *aarF*, *dfp*, *galR*, *glnS*, *hemA*, *rfaE*, and *speA*. In addition, we obtained markers from Achtman et al. (1999) (*dmsA*, *glnA*, *manB*, *thrA*, *tmk*, and *trpE*) and from Revazishvili et al. (2008) (*16S rDNA*, *gyrB*, *yhsp*, *psaA* and *recA*). We created a custom BLAST (Altschul et al., 1990) database with our new genome sequences combined with the publicly available genomes for all three species groups.

We created datasets based on SNPs by searching for *k-mer* = 25 (SNP on position 13) among unaligned genomes, as implemented in kSNP 2.0 (Gardner & Hall, 2013; Gardner & Slezak, 2010). We chose this implementation because it does not depend on arbitrarily selecting a reference genome, it can take draft and unassembled sequence data (including low-coverage genomes), and it is fast and widely used in epidemiological studies (Epson et al., 2014; Pettengill et al., 2014; Raphael et al., 2014; Timme et al., 2013). Briefly, the optimal *k-mer* size was estimated using Kchooser, which identifies threshold value of *k* for which non-unique *k-mers* are the result of real genome redundancy, not chance. We kept all SNPs that were shared among all taxa in a given dataset (core SNP subset), which were used to build matrices for downstream analyses. The matrices used contained only variable bi-allelic sites from non-overlapping *k-mers* and their size is described in Table 2.

**Table 2:** Genetic diversity and dataset length for different species and molecular survey approaches.

		MLST		SNP				Genome			
				Chromosome I		Chromosome II		Chromosome I		Chromosome II	
		Mean	Variance	Mean	Variance	Mean	Variance	Mean	Variance	Mean	Variance
**Bp**	theta	36.05	127.39	7807.03	5593613.54	4333.68	1724029.33	4666.60	1999005.02	2187.99	439709.21
	pi	4.45E–03	5.15E–06	9.58E–02	2.18E–03	9.69E–02	2.24E–03	5.67E–03	7.66E–06	7.14E–03	1.21E–05
	length	3518.00		31189.00		17313.00		289172.00		108654.00	
**Br**	theta	112.58	1205.64	914.77	78109.73	234.25	5161.72	635.85	37782.92	2046.37	390305.58
	pi	1.00E–02	2.46E–05	8.01E–02	1.54E–03	7.66E–02	1.43E–03	7.96E–03	1.52E–05	1.62E–02	6.25E–05
	length	4409.00		3628.00		929.00		24110.00		36223.00	
**Yp**	theta	36.17	120.42	3204.65	883301.99			527.40	24020.48		
	pi	4.97E–04	6.68E–08	5.60E–02	7.40E–04			4.55E–04	4.94E–08		
	length	20498.00		14116.00				281149.00			

**Notes.**

Bp
*Burkholderia pseudomallei*
Br
*Brucella spp.*
Yp
*Yersinia pestis*

We created full genome datasets by aligning complete genome sequences in Mauve 2.3.1 (Darling, Mau & Perna, 2010) and then used the resulting multiple sequence alignment directly and/or reduced for phylogenetic inference. The reduced full genome dataset consisted of all Locally Collinear Blocks (LCBs) detected by Mauve that were greater than 10 Kb and randomly subsampled up to a total of 300 Kb present across all taxa in a given dataset.

### Diversity and phylogenetic analyses

We measured genetic diversity as the substitution rate-scaled effective population size Θ for all molecular survey approaches (MLST, SNP, WGS), as implemented in the ‘pegas’ package in R (Paradis, 2010). We inferred phylogenies, both with and without assuming a molecular clock. Clock phylogenies were inferred using Bayesian Inference (BI) and Markov Chain Monte Carlo (MCMC) simulations as implemented in Beast 1.7.5 (restricting the analysis to those sequences with recorded dates) and using the Beagle library to speed up analysis (Ayres et al., 2012; Drummond et al., 2012). We assumed a General Time Reversible (GTR) substitution model for all three data approaches with a discrete gamma distribution (4 categories) to model rate heterogeneity (MLST datasets were partitioned by gene with a model fit per gene; rate heterogeneity was not modeled for SNP datasets). We unsuccessfully tried to partition the genome dataset by gene, but phylogenetic inference did not reach convergence. Briefly, MCMC simulations were run until a single chain reached convergence, as diagnosed by its trace and ESS values (>400; ranging from 2E^8^ to 2E^9^ steps; 10% burnin) in Tracer 1.5 (http://tree.bio.ed.ac.uk/software/tracer/) and tree distributions were summarized in TreeAnnotator 1.7.5 (10–20% of trees were discarded as burnin). The molecular clock (strict clock model) was calibrated using isolate collection dates and a uniform distribution (from 0 to 1) as clock prior. We also used BI for non-clock phylogenies as implemented in MrBayes 3.2 (Ronquist et al., 2012) where we ran 8 chains (6 heated), 2E^7^ generations each. As in the clock phylogenies, we used visual inspection of the traces as well as the average standard deviation of split frequencies to assess convergence. All trees were rooted by using outgroups (*Yersinia pseudotuberculosis* IP31758, *Ochrobactrum anthropic*, and *Burkholderia thailandensis* E264).

In order to compare tree topologies, we applied two topology metrics, Robinson–Foulds (RF, Robinson & Foulds, 1981) and Matching Splits Clusters (MC, Bogdanowicz & Giaro, 2012) to compare topologies across different molecular survey approaches and among chromosomes as implemented in TreeCmp (Bogdanowicz & Giaro, 2012). We also assessed the extent to which phylogenies and traits (host range, sample collection site, and sampling date) were correlated through Bayesian Tip-Significance testing by estimating the Association Index (AI, Wang et al., 2001) and Parsimony Score (PS, Slatkin & Maddison, 1989) as implemented in BaTs (Parker, Rambaut & Pybus, 2008). Figures were plotted using ggplot2 (Wickham, 2009) and APE (Paradis, Claude & Strimmer, 2004) packages, and high posterior density (HPD) intervals were estimated using TeachingDemos package (Snow & Snow, 2013).

## Results and Discussion

Sequencing technologies and statistical phylogenetic methods are arming researchers with powerful tools to track infectious agents over space and time with unprecedented resolution (Holt et al., 2012; Lewis et al., 2010). However, with multiple molecular survey approaches and a battery of analytical methods, it is not clear how these interact.

Using 115 genomic sequences (60 this study + 55 GenBank), we compared inferences regarding genetic diversity, substitution rates and node ages, tree topologies, structure and phylogenies inferred from different molecular survey approaches. We use the term “molecular survey approaches” to refer to either MLST, SNP, or WGS approaches, and the term “species datasets” or simply “dataset” to refer either to *B. pseudomallei*, *Brucella spp*. or *Y. pestis* sequence data belonging to any of these species/genera. Given the difficulty of current algorithm implementations in reading and analyzing whole bacterial genomes, we decided to randomly sub-sample core homologous regions to compile genomic data that we termed “genome” (see Methods for details).

### Diversity and datasets

Datasets sizes varied in length by data approach, species, and genomic partition (chromosome I/II). Notably, we intended to include as many genes as possible for the MLST schemes, which resulted in partitioned datasets ranging from 7 to 18 genes. In the case of *Y. pestis*, the MLST dataset constituted a larger dataset than the SNP dataset due to the low variability in this species. The interspecific dataset, i.e., *Brucella spp.*, rendered the smallest dataset for all data approaches (least number of sites) as opposed to intraspecific datasets (*Y. pestis*; *B. pseudomallei*) that ended up being one or two orders of magnitude longer (Table 2; square brackets).

In order to characterize the present genetic diversity of our datasets, we estimated effective population size using a segregating sites model (Θ; Watterson’s theta) and nucleotide diversity (*π*) (Nei, 1987; Paradis, 2010; Watterson, 1975). Nucleotide diversity ranked higher for SNPs compared to other approaches for the same species, as these data only contain binary variable sites (Table 2). Nucleotide diversity was higher for *B. pseudomallei* than for *Brucella spp*. and *Y. pestis* when SNP data were analyzed. However, this was not observed for either MLST or genome data, where nucleotide diversity ranked higher for *Brucella spp.* compared to *B. pseudomallei*. *Y. pestis* nucleotide diversity was consistently lower compared to other datasets across molecular approaches. Θ estimates were higher for *B. pseudomallei* than others for SNP and genome data, but not MLST data where *Brucella spp*. yielded the larger Θ (Table 2).

### Rates and ages

**Figure 1:**
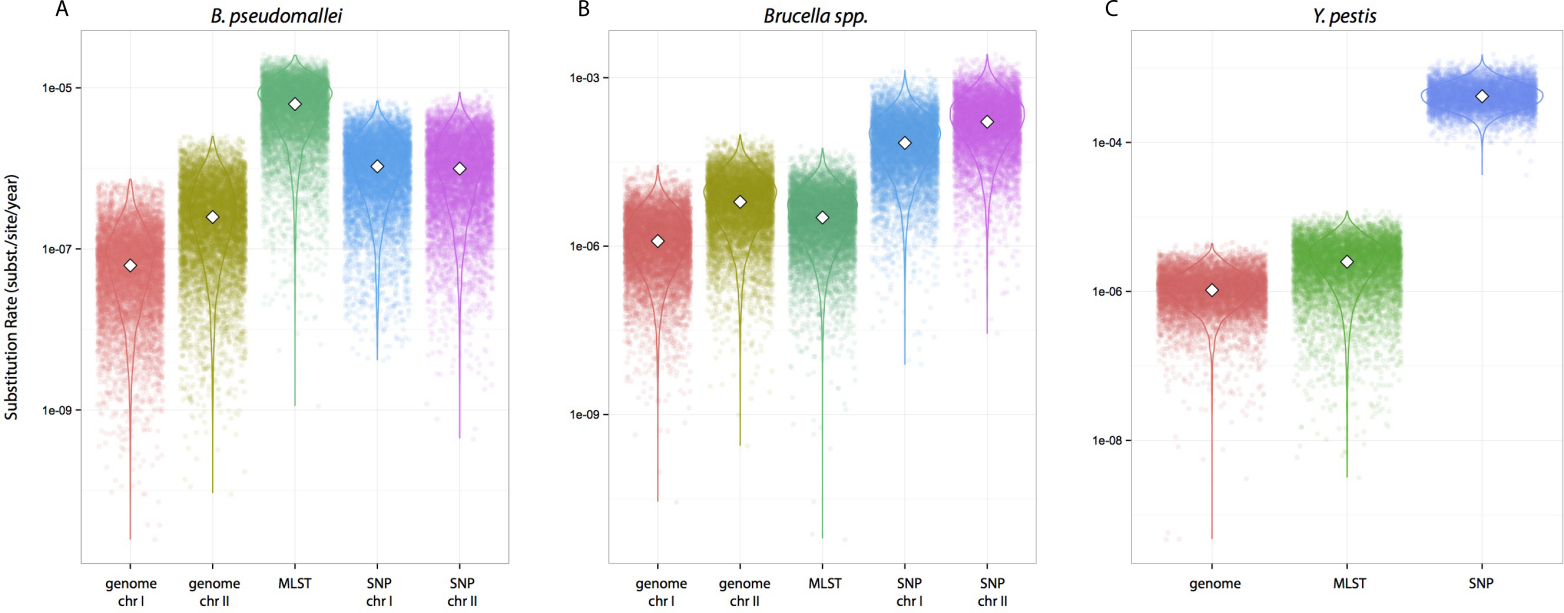
Substitution rates for all datasets as estimated from different molecular survey approaches. Genome/SNP chr I/II refers to estimates from different chromosomes. *Burkholderia pseudomallei* (A), *Brucella spp.* (B), and *Yersinia pestis* (C). Note different scale for species rates.

We tested whether different data approaches resulted in different inferences regarding substitution rates and node ages while maintaining other parameters constant, i.e., clock calibrations, substitution models, tip dates, coalescent tree priors, and taxa (different partition scheme; see Methods for details). Substitution rate estimates were always higher for SNP data compared to genome data, irrespective of species datasets used (Fig. 1). Rates estimated from MLST data were largely overlapping with estimates from genome data for *Y. pestis* and *Brucella spp.* including median values (highlighted in Figs. 1B–1C). However, this was not the case for the *B. pseudomallei* dataset where, although the distributions overlapped, median values for the substitution rate estimate from MLST data were higher by at least an order of magnitude compared to estimates from other approaches (MLST rate median = 6.30E^−7^; genome chr I = 6.17E^−8^; genome chr II = 2.48E^−7^; SNP chr I = 1.06E^−6^; SNP chr II = 9.94E^−7^ [rates in substitutions per site per year]).

**Figure 2:**
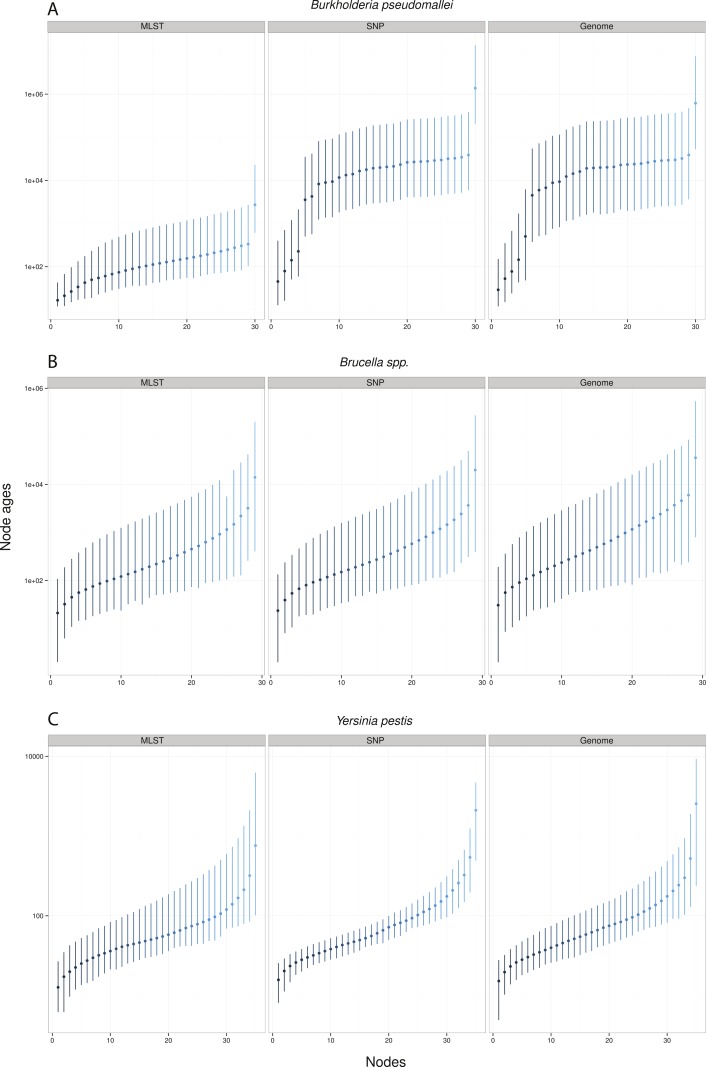
Median node ages in years. *Burkholderia pseudomallei* (A), *Brucella spp.* (B), and *Yersinia pestis* (C) median estimates and their 95% highest posterior density (HPD) interval according to molecular survey approach (only chromosome I showed; see Fig. S1). Nodes are numbered from youngest to oldest.

Remarkably, when collecting node ages and comparing them across data approaches, we found that highest posterior density intervals (95% HPD) overlapped substantially in the case of *Y. pestis* and *Brucella spp.* datasets (Figs. 2B–2C). We observed the same trend with SNP and genome approaches when analyzing *B. pseudomallei* datasets, but not with MLST data (Fig. 2A). Interestingly, in *Y. pestis* node age estimates we observed that 95% HPD intervals were narrower in SNP and genome data than in MLST data. This suggests that, though different molecular survey approaches result in markedly different substitution rate estimates, node ages 95% HPD are largely overlapping and thus not significantly different.

Substitution rate estimates differ substantially (up to 2 orders of magnitude), though their posterior distributions overlap to various degrees. Generally, substitution rate estimates drawn from SNP data were higher than those from MLST and genome data. However, node ages are largely consistent across molecular survey approaches, especially for *Brucella spp.* data (interspecific and intermediate diversity dataset). This supports the practice of using SNP data coupled to Bayesian inference coalescent methods to infer divergence times, even though traditional reversible substitution models are not specifically designed for this molecular approach. Substitution models based on models for discrete morphological character changes have been suggested, but are not widely popular (Lewis, 2001).

### Phylogenies and topology comparisons

**Figure 3:**
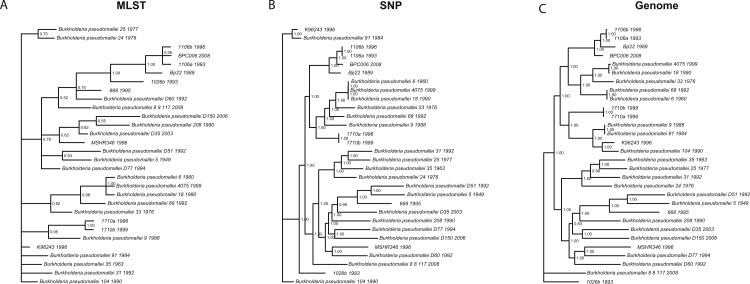
*Burkholderia pseudomallei* phylogenies by survey approach. MLST phylogeny (A) is less resolved and poorly supported compared to SNP (B) and genome (C) phylogenies (only chromosome I showed).

We wanted to determine whether different data approaches produce different phylogenies and to quantify the extent of any observed differences in topology (dataset sizes in Table 2). We inferred phylogenies for every species under all three molecular survey approaches, partitioned by chromosome when appropriate, without assuming a molecular clock and outgroup rooted (see ‘Methods’). We used two topology metrics, Matching Clusters (MC), rooted version of Matching splits (Bogdanowicz & Giaro, 2012), and R–F Clusters (RC), rooted version of Robinson–Foulds metric (Bogdanowicz, Giaro & Wróbel, 2012; Robinson & Foulds, 1981). MC distances reflect the minimal number of cluster (or clade) movements needed so that the two phylogenies are topologically equivalent. RC distances measure the average number of cluster differences between two phylogenies. Likewise, MC distances can be interpreted as reflecting changes deep in a phylogeny, and RC distances, in turn, as reflecting changes at the tip of the phylogenies or for more recent relationships. In general, we found that the phylogenies inferred using MLST data are less resolved and more poorly supported (posterior probabilities) than those inferred by either SNP or genome data for all species datasets (Fig. 3 and Fig. S3). This is also reflected in MC distances, where topologies inferred by MLST data are as distant, or more so, to SNP/genome based topologies than between SNP and genome topologies, with some exceptions (Table 3).

**Table 3:** Topology distances among phylogenies inferred using different molecular survey approaches. Bolded rows show tree comparisons between different chromosomes under the same molecular survey approach.

Species	Tree comparisons		Matching cluster	R–F cluster
*B. pseudomallei*	mlst	snp-I	181	16
*B. pseudomallei*	mlst	snp-II	162	17
*B. pseudomallei*	mlst	genome-I	149	18
*B. pseudomallei*	mlst	genome-II	116	18
***B. pseudomallei***	**snp-I**	**snp-II**	**33**	**7**
*B. pseudomallei*	snp-I	genome-I	56	17
*B. pseudomallei*	snp-I	genome-II	91	17
*B. pseudomallei*	snp-II	genome-I	47	19
*B. pseudomallei*	snp-II	genome-II	72	16
***B. pseudomallei***	**genome-I**	**genome-II**	**63**	**14**
*Brucella spp.*	mlst	snp-I	34	5
*Brucella spp.*	mlst	snp-II	10	1.5
*Brucella spp.*	mlst	genome-I	73	4.5
*Brucella spp.*	mlst	genome-II	56	5
***Brucella spp.***	**snp-I**	**snp-II**	**24**	**5.5**
*Brucella spp.*	snp-I	genome-I	61	5.5
*Brucella spp.*	snp-I	genome-II	40	5
*Brucella spp.*	snp-II	genome-I	63	5
*Brucella spp.*	snp-II	genome-II	50	4.5
***Brucella spp.***	**genome-I**	**genome-II**	**67**	**7.5**
*Y. pestis*	mlst	snp	223	13.5
*Y. pestis*	mlst	genome	103	8
*Y. pestis*	snp	genome	124	8.5

**Notes.**

Genome/SNP-I/II, chromosome I or II; R–F Cluster, Robinson–Foulds for rooted trees metric.

For *Brucella spp*. and *Y. pestis*, MC distances are clearly higher between SNP/genome and MLST; however, RC distances do not follow this trend. Since MC metric concentrates more on differences corresponding to branches deep in the topologies as opposed to RC, these results suggest that SNP and genome topologies have more similar backbones when compared to each other than MLST topologies. Likewise, MLST topologies are more similar at the tips rather than deep in the topologies (Fig. 3 and Table 3). Of course, to determine which approach is more accurate would require a dataset of known evolutionary history, but SNP and genome approaches appear to be more consistent with one another, especially for the deeper nodes.

Slowly evolving pathogens can be difficult to track as their populations accrue fewer substitutions, and/or genomic changes may or may not reflect ecological processes, such as host switches or geographic spread (see below for association testing). For instance, phylogenies inferred using MLST data were less resolved and poorly supported compared to their SNP and genome counterparts, even though in some cases (e.g., *Brucella* spp./*Y. pestis*) the MLST dataset size was larger than the SNP size dataset. This argues for the need to acquire genome data, as those data constitute the ultimate source of genealogical information, especially when analyzing monomorphic or clonal species, i.e., *Y. pestis* (Achtman, 2008; Achtman et al., 1999). We also found that strongly supported phylogenies, e.g., those based on SNP and genome data, can support conflicting hypotheses and thus will be misleading. For instance, *B. pseudomallei* clades, including isolates 1106a, 1106b, Bp22, and BPC006, all show posterior probabilities = 1, yet their relationships differ, hence a caveat when analyzing SNP/genome data and drawing conclusions about relationships amongst isolates.

### Phylogenetic associations with geography, time, and host

Phylogenetic inference often is performed to infer ecological processes that leave a genomic imprint. Phylogeny-trait associations are essential to elucidate these processes. Accordingly, we estimated the Association Index (AI), and Parsimony Score (PS) on three traits (sampling location, sampling time, and host), and tested whether different answers were obtained by molecular survey. Results for *B. pseudomallei* showed significant association with sampling location and sampling time, but not with host for most of the datasets (AI and PS; Table 4). Likewise, *Y. pestis* datasets were significantly associated with sampling location and, to some extent, with sampling time and host. Interestingly, *Brucella spp.* showed significant genetic structure to be associated with both sampling location and host, but not sampling time (Table 4).

**Table 4:** Trait-phylogeny association statistics. Significant associations (*p* value <0.05) were found between traits (sampling location/host/time) and phylogenies inferred by using different data approaches. Association index (AI); Parsimony Score (PS); genome/SNP-I/II, chromosome I or II.

*Statistic*	*Trait*		
	**Sampling location**		
	*B. pseudomallei*	*Brucella spp.*	*Y. pestis*
**AI**	MLST, genome-I, genome-II, SNP-I, SNP-II	MLST, genome-I, genome-II, SNP-I, SNP-II	MLST, genome, SNP
**PS**	MLST, genome-I, genome-II, SNP-II	MLST, SNP-II	MLST, genome, SNP
	**Host**		
	*B. pseudomallei*	*Brucella spp.*	*Y. pestis*
**AI**	None	MLST, genome-I, genome-II, SNP-I, SNP-II	Genome, SNP
**PS**	None	MLST, genome-I, genome-II, SNP-I, SNP-II	None
	**Time**		
**AI**	Genome-I, genome-II	None	Genome
**PS**	None	None	Genome

Irrespective of the molecular survey approach used, phylogenies derived from *B. pseudomallei* showed a significant association with sampling location but not with host, suggesting similar evolutionary forces acting on *B. pseudomallei* in different hosts, or that *B. pseudomallei* isolates are highly endemic to the sites from which they were isolated. Similarly, *Brucella spp.* phylogenies were associated with both sampling location and host, irrespective of the data approach used, which most likely reflects metabolic and geographic constraints on gene flow. Interestingly, for *Y. pestis*, no significant association of host and MLST data was observed, which most likely reflects lack of signal, given the absence of resolution of phylogenies in its posterior distribution.

Molecular survey approaches have different sets of assumptions and properties that must be considered before an analysis is done. Similarly, statistical models that are employed may be suited for certain data approaches and not others. Here, we used popular phylogenetic methods for all molecular approaches to test whether congruent inferences could be obtained, even though some might violate particular model assumptions. The MLST method targets housekeeping genes that are likely to be maintained across taxonomic levels, hence amenable for evolutionary inferences. Yet, similar to other molecular survey approaches, they are likely to be subjected to selective pressures, which may or may not impact evolutionary inferences because of molecular convergence (Castoe et al., 2009; Edwards, 2009) and estimation of branch lengths (Ho et al., 2011; Roje, 2014). Other trade-offs of MLST have been discussed elsewhere, mainly with respect to utility and how they can be refashioned in the post-genomic era (Maiden et al., 2013; Pérez-Losada et al., 2013). On the other hand, sampling bias can influence phylogenetic analysis (Lachance & Tishkoff, 2013). Here, we obtained SNP data without using reference data and included globally sampled genomes and stringent quality controls (high Phred scores, long *k-mers*) to diminish ascertainment and discovery bias (Gonçalves da Silva et al., 2014). However, standard nucleotide substitution models, such as GTR, are not designed to account for binary sites-only datasets nor Bayesian Inference methods, which typically factor in invariable sites, influencing branch length estimation and impacting parameter estimates such as substitution rate and divergence time. Nonetheless, they have been used to date the spread of bacteria and other pathogens (Comas et al., 2013; Holt et al., 2010; Holt et al., 2012; Okoro et al., 2012; Pepperell et al., 2013). We speculate, based on these results, that analysis of SNP data to survey genomic variation is robust and can produce inferences that are not substantially different from WGS data. However, a recent study using simulated data has shown that using SNPs and a single reference introduce systematic biases and errors in phylogenetic inference (Bertels et al., 2014).

## Conclusions

The field of bacterial population genomics is advancing rapidly with larger datasets (more taxa, more sites) increasingly available, including whole-genomes, making greater resolution possible and more powerful exploration of complex issues (Chewapreecha et al., 2014; Nasser et al., 2014). The results of analyses reported here show that the molecular survey that is used can have a critical impact on substitution rate and phylogenetic inference. However, node dates and trait associations are relatively consistent irrespective of the survey tool used. We found substitution rates vary widely depending on the approach taken, and SNP and genomic datasets yield different, but strongly supported phylogenies. Overall, inferences were more sensitive to molecular survey in the low diversity *Y. pestis* dataset, compared to the *B. pseudomallei* and *Brucella spp.* datasets.

Substitution rate estimates are important because, coupled to sampling dates, they allow tracking infections in space and time, and thus provide an essential epidemiological tool for monitoring and control of infectious diseases. The results presented strongly suggest that future studies should consider discordances between inferences derived from different molecular survey methods, especially with respect to substitution rate estimates.

In practice, other variables also influence what type of survey approach to use, and there are foreseeable cases where it might be practical to choose for MLST over WGS/SNPs, e.g., cost, equipment, ease of use, and necessary expertise. More importantly, coupling multiple molecular survey approaches could be useful in gaining biological insights (e.g., genome evolution, gene synteny, and content using WGS) and genotyping large numbers of samples using MLSTs.

Importantly, for whole genome analysis, a subset of data is selected to run existing software to estimate population genetic parameters. Clearly, there is a need to expand the range of methods to include whole genome data analysis. However, as bacterial genomics matures, current methods will need to be modified and extended to handle the stream of data now being generated.

## Supplemental Information

10.7717/peerj.761/supp-1 Figure S1 Median node ages for *Burkholderia pseudomallei *(A), *Brucella spp. *(B)

10.7717/peerj.761/supp-2 Figure S2 Phylogenies by molecular survey approach. *Brucella *spp. phylogenies (A) and Y. pestis phylogenies (B)

10.7717/peerj.761/supp-3 File S1 SRA Accession numbers for all 60 genomes contributed in this study

10.7717/peerj.761/supp-4 Map S1 Supplemental Map Geographic distribution of isolates used in this study

10.7717/peerj.761/supp-5 File S2 Datasets, Clock and non-clock phylogenies for all molecular survey approaches and species

## Additional Information and Declarations

### DNA Deposition

The following information was supplied regarding the deposition of DNA sequences: GenBank: SRP022877; SRP022862; SRP023117.

### Data Deposition

The following information was supplied regarding the deposition of related data: All supplemental information has been deposited in FigShare at: http://dx.doi.org/10.6084/m9.figshare.1091392.
