# Supplementary figures and images for: Concordance and discordance of sequence survey methods for molecular epidemiology
Source: PeerJ. 2015 Feb 17;3:e761. doi: 10.7717/peerj.761 (PMC4338773; doi:10.7717/peerj.761)

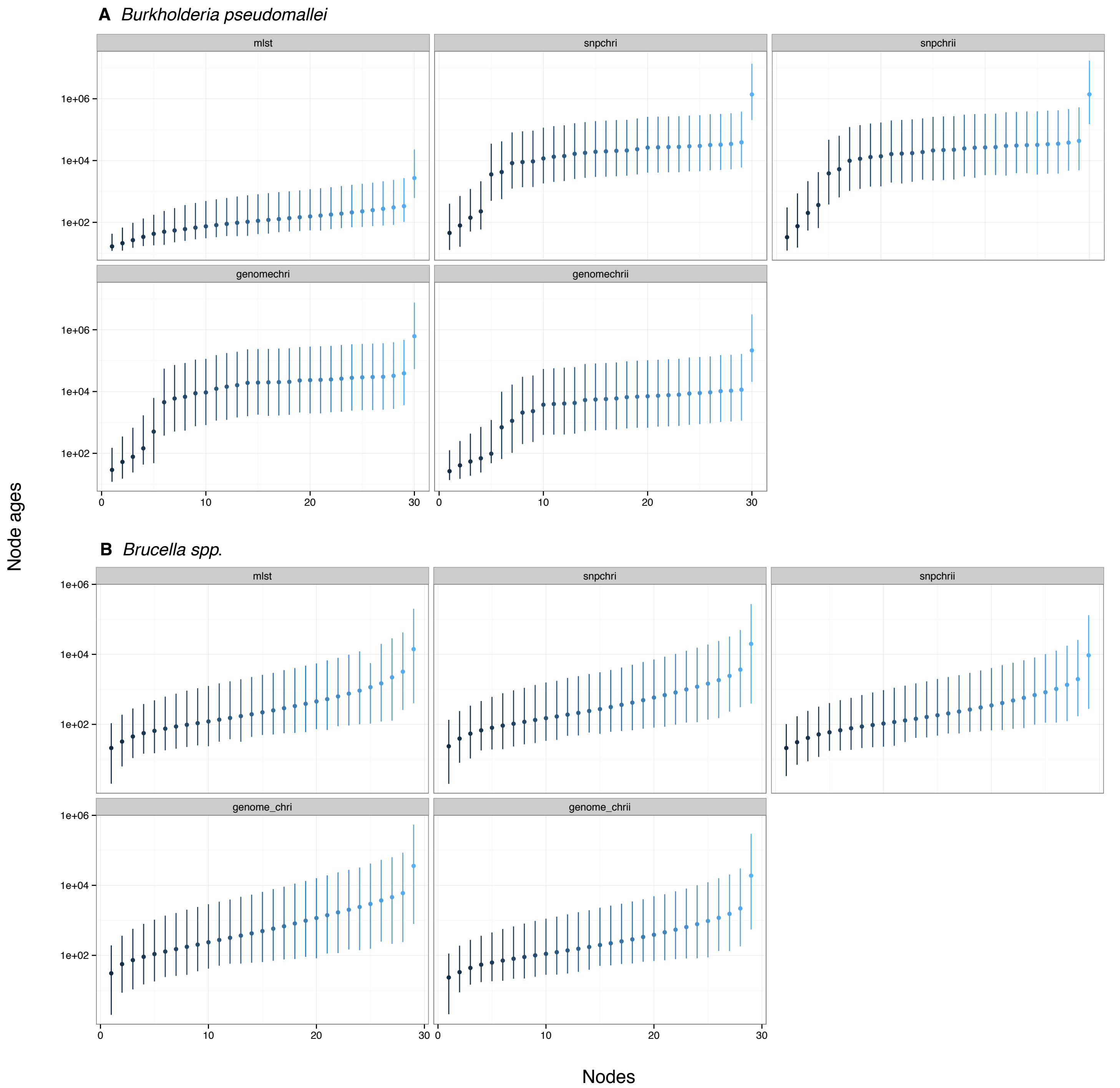

Supplement: Figure S1 [file peerj-03-761-s001.pdf]

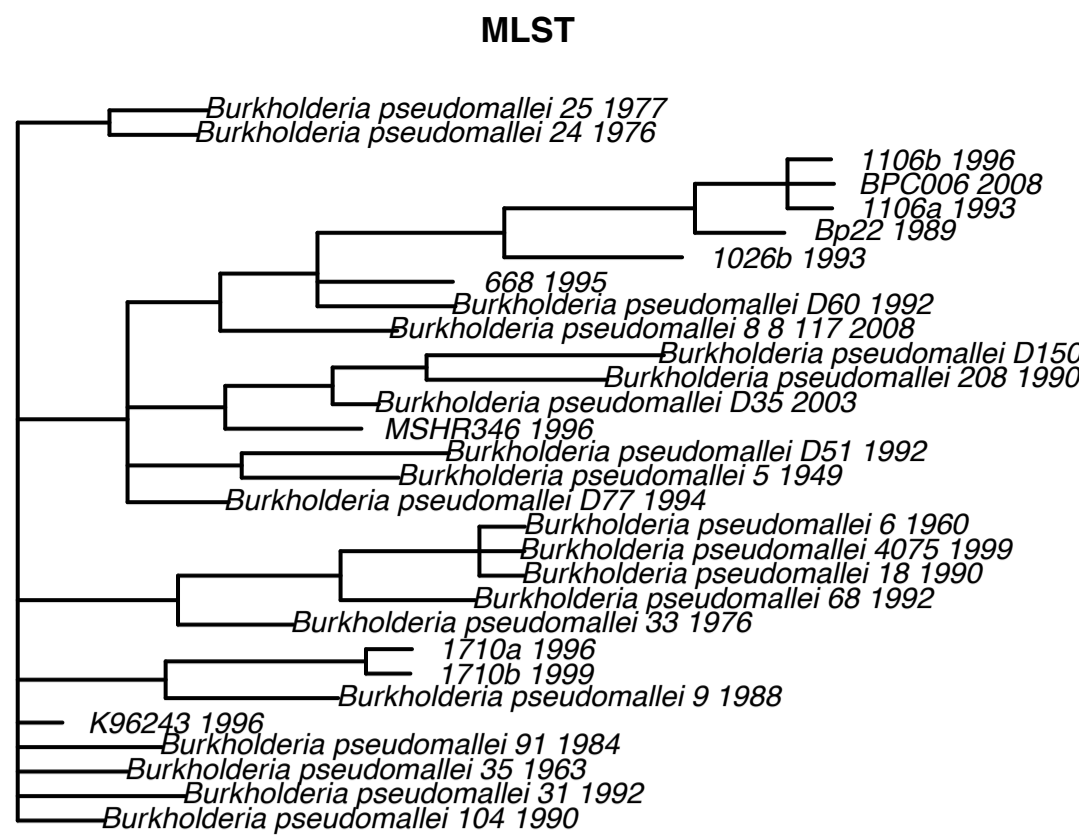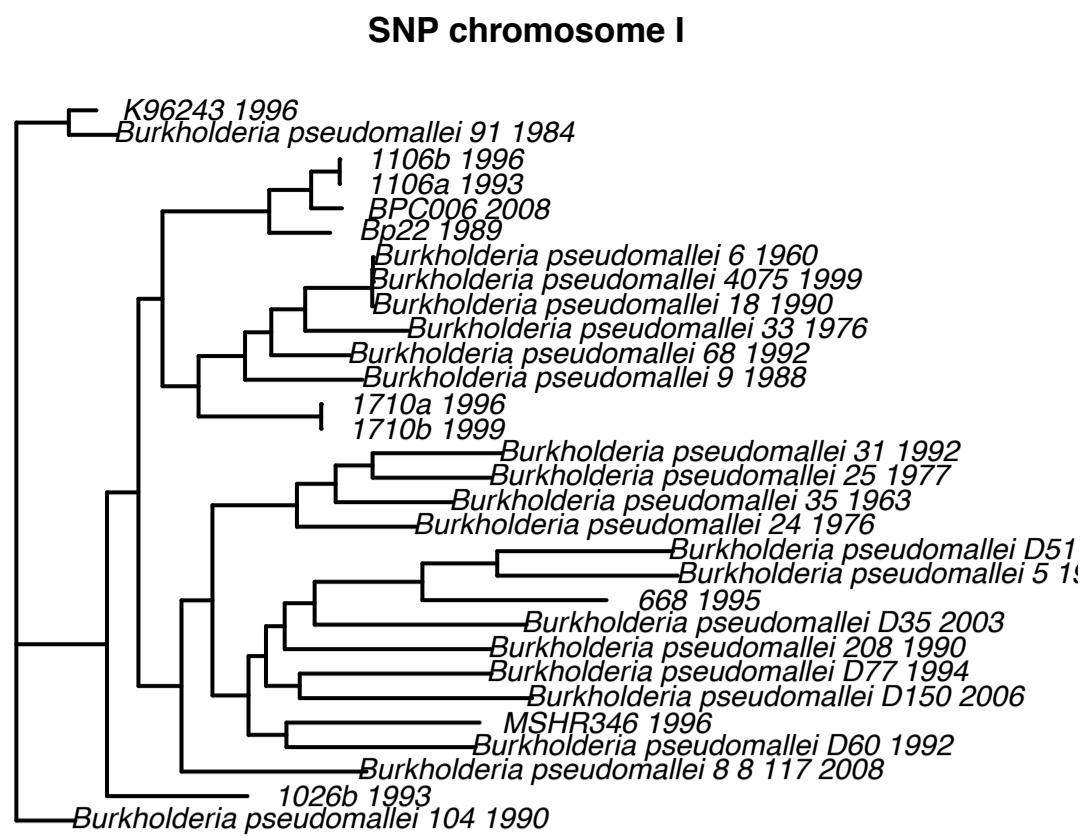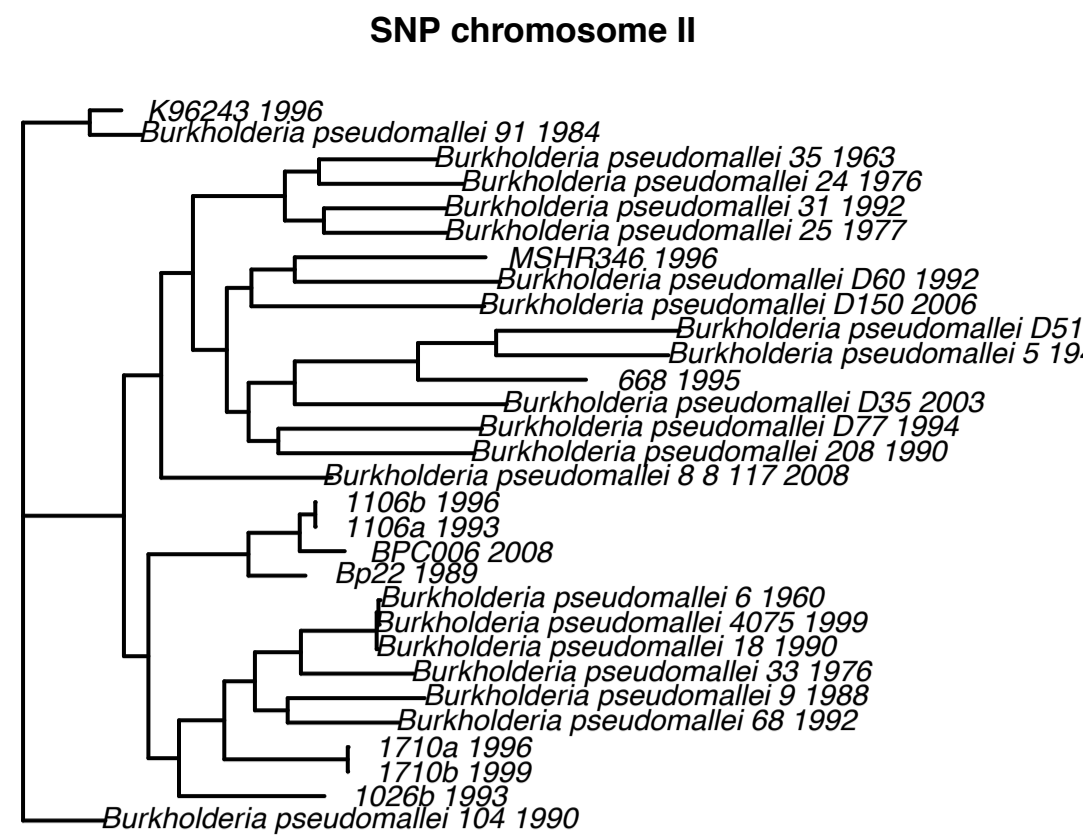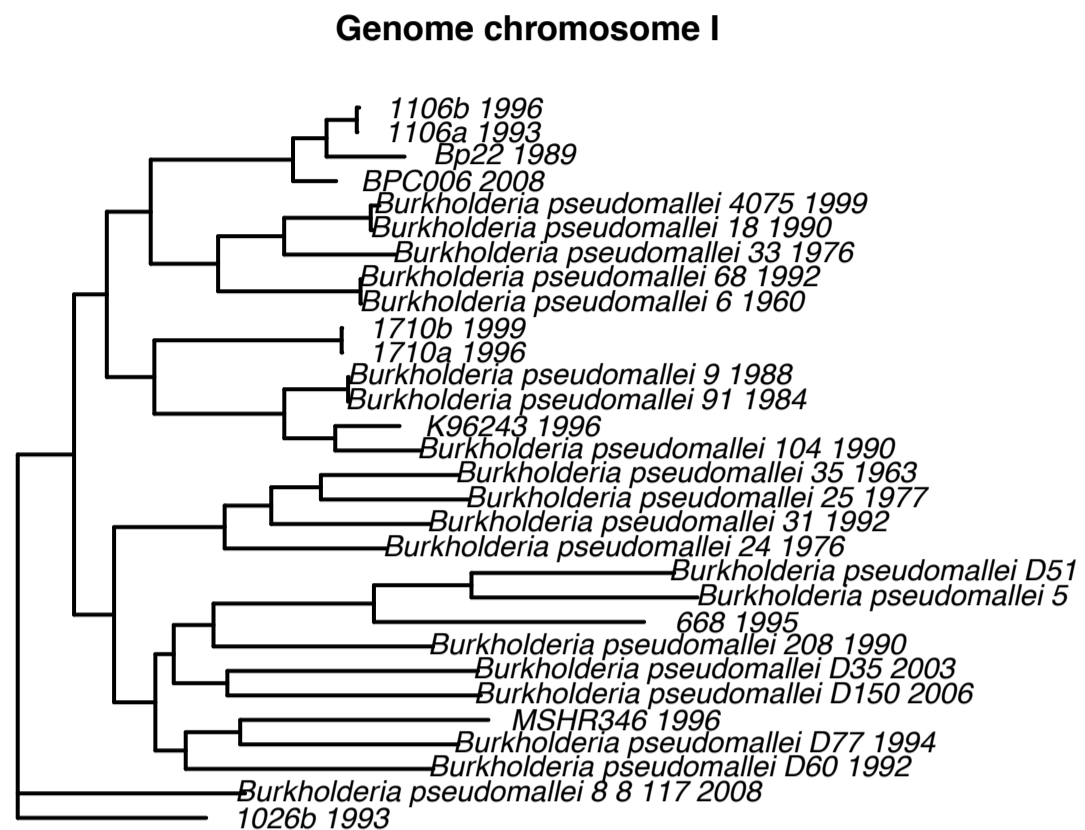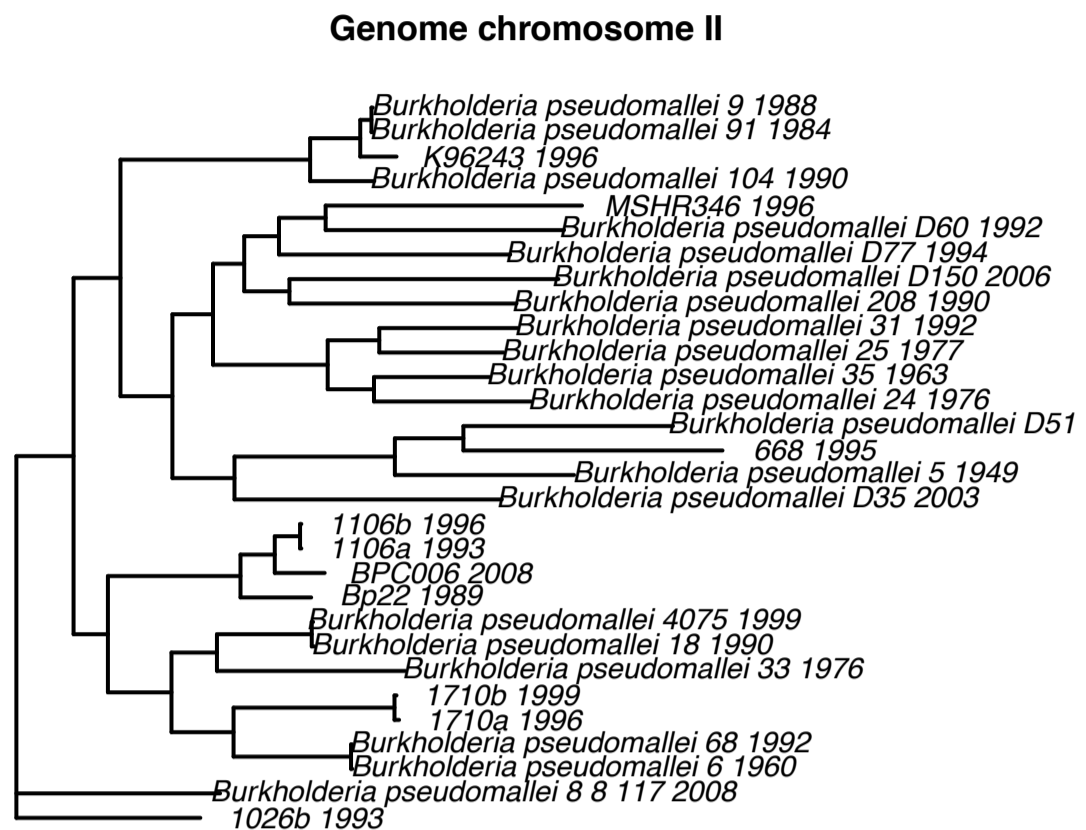

### A *Burkholderia pseudomallei*

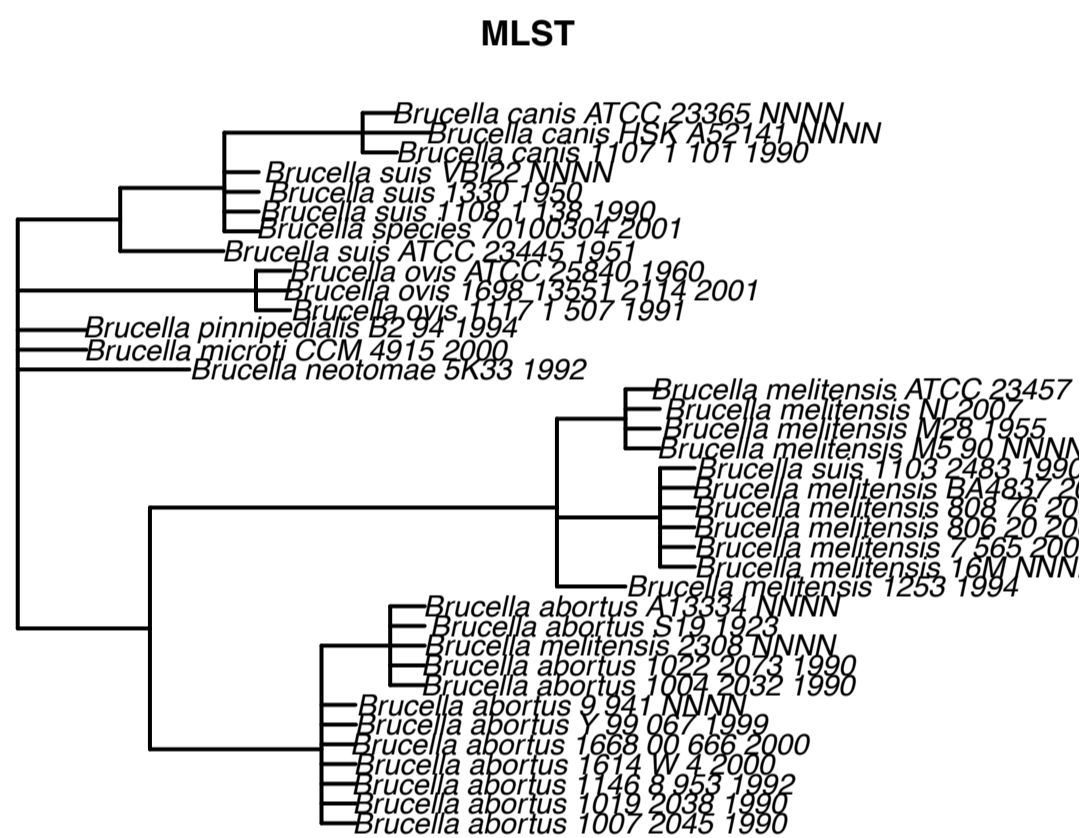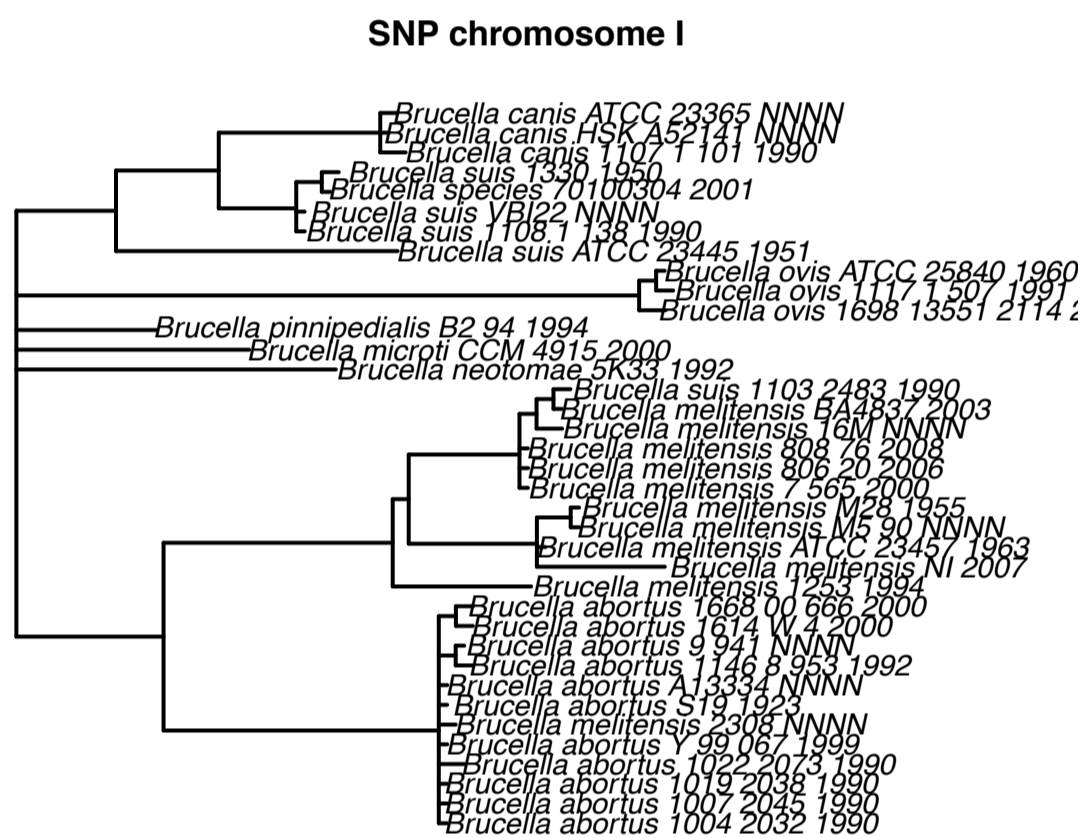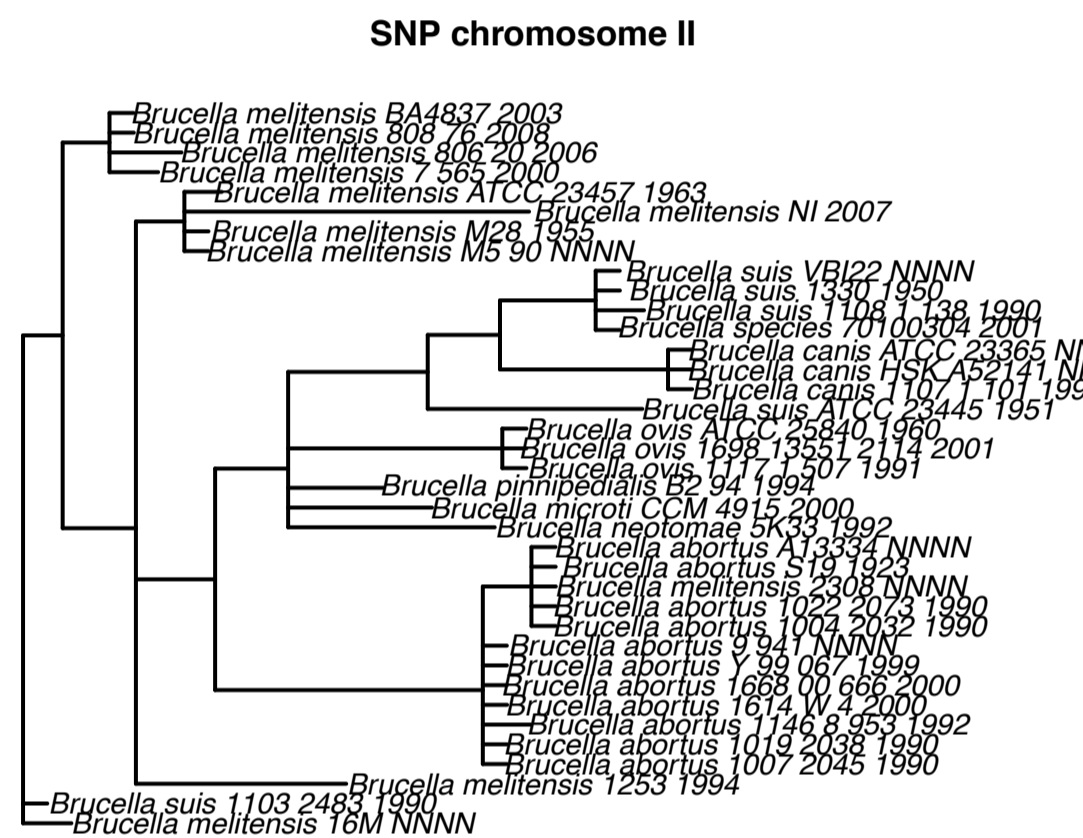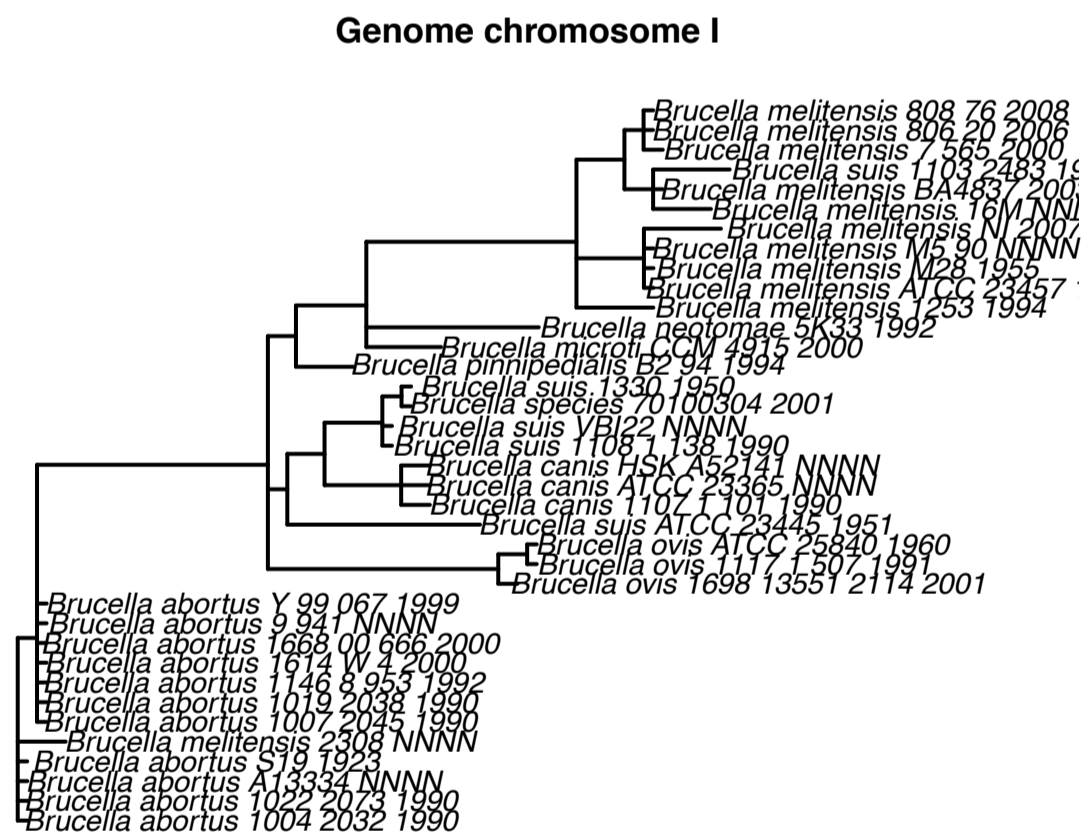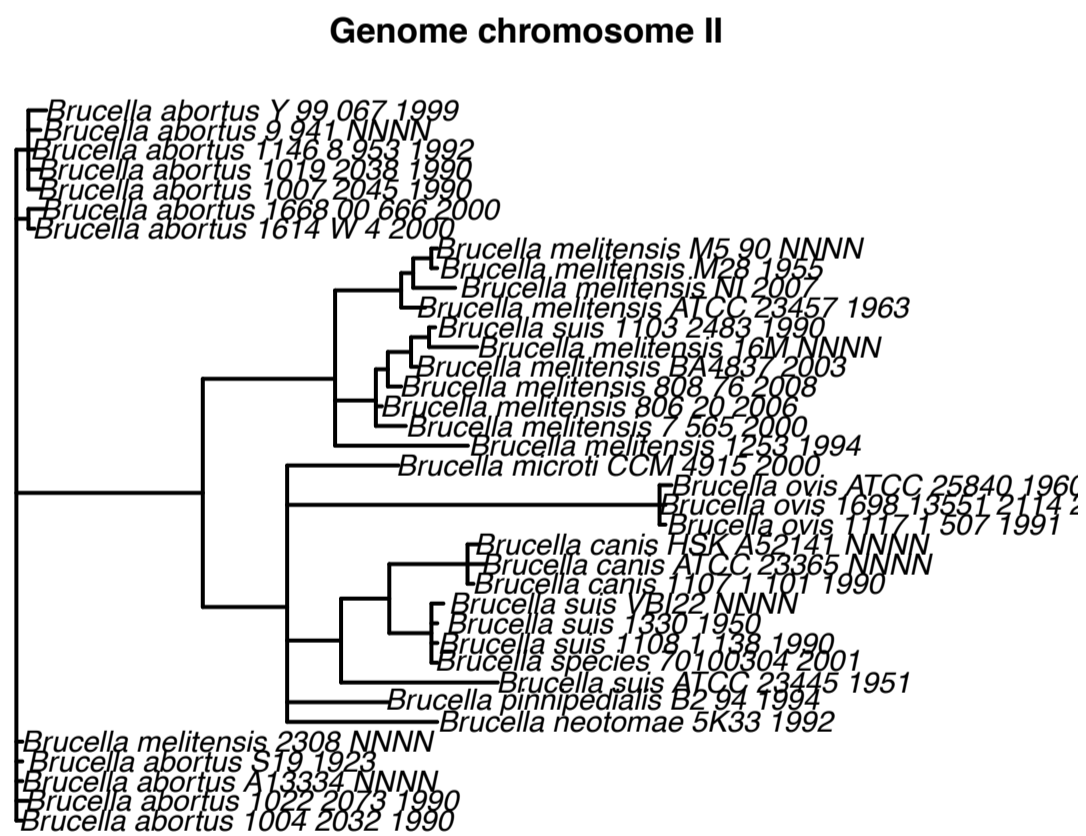

**B** *Brucella* spp.

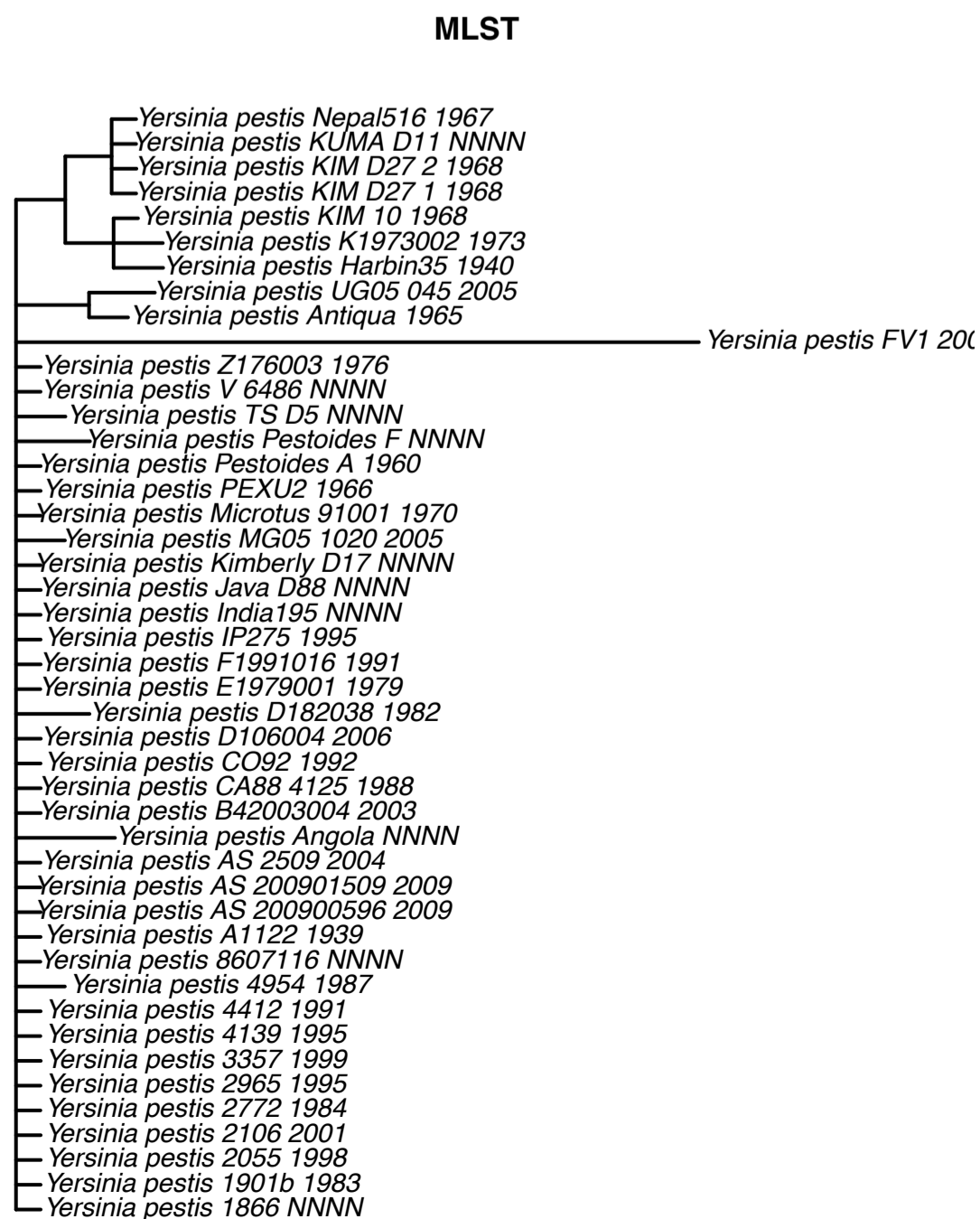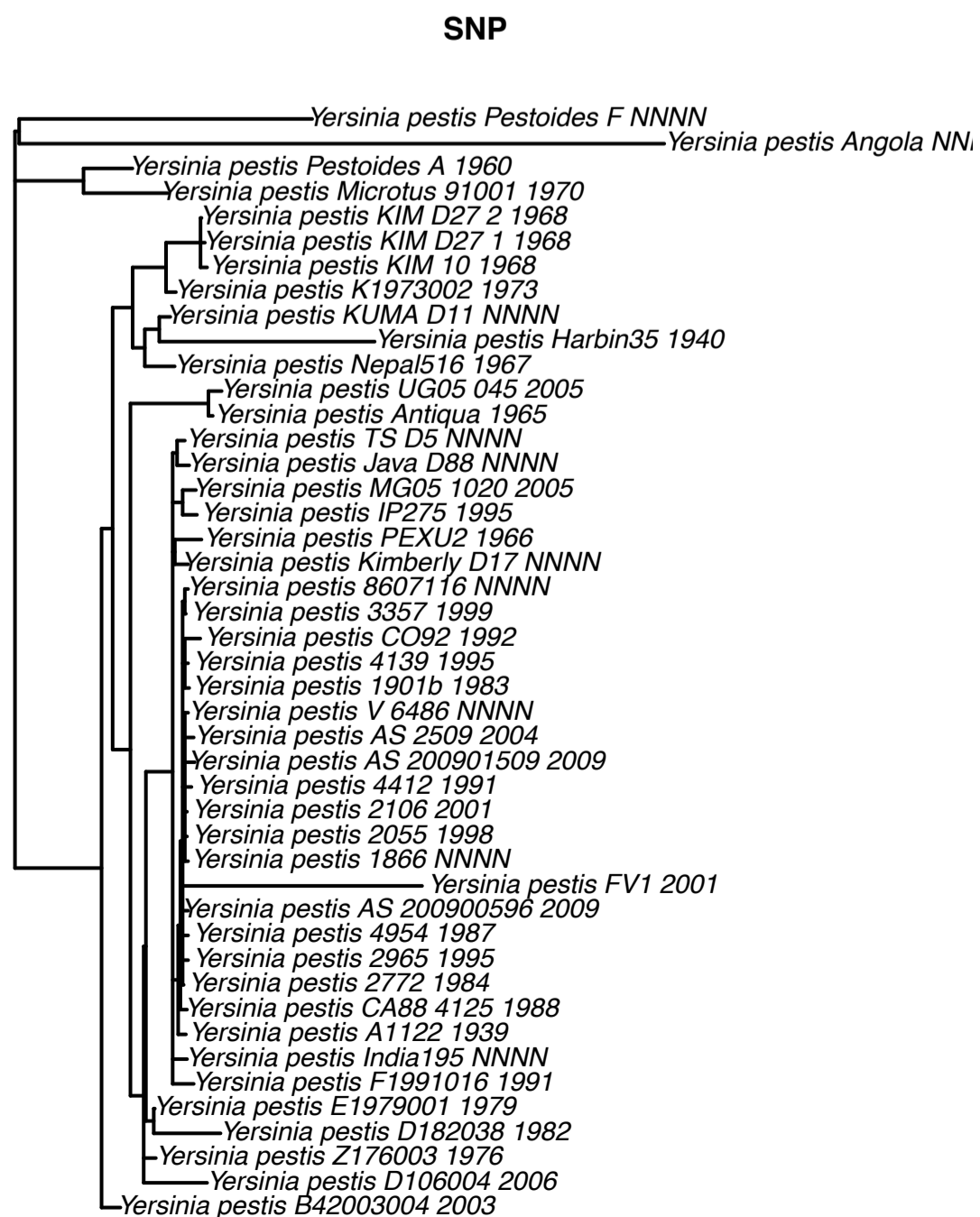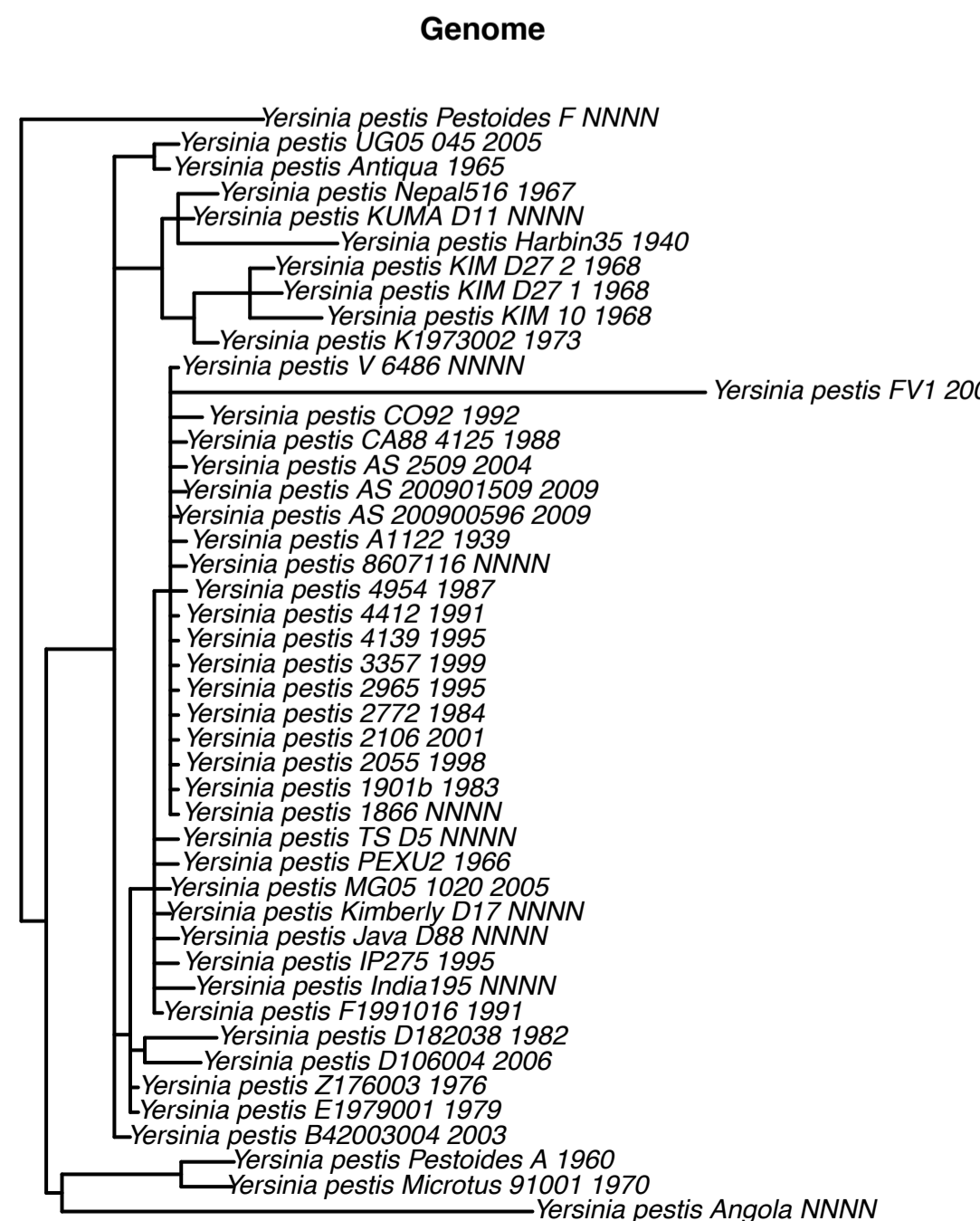

### C *Yersinia pestis*

Supplement: Figure S2 [file peerj-03-761-s002.pdf]

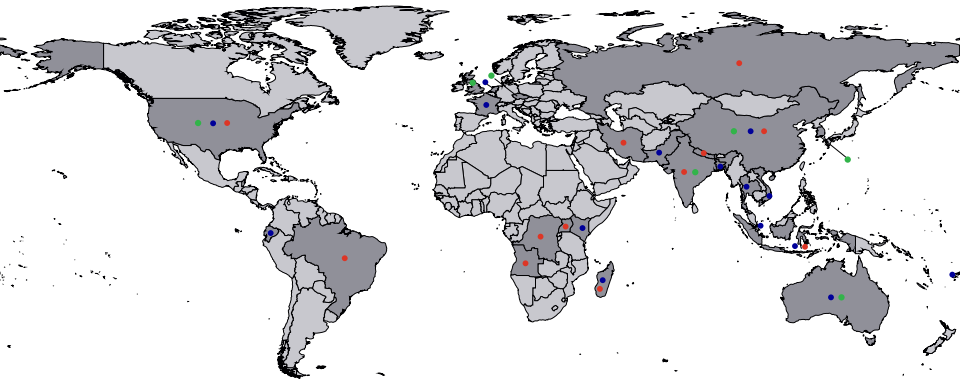

- *Burkholderia pseudomallei*
- *Brucella* spp.
- *Yersinia pestis*

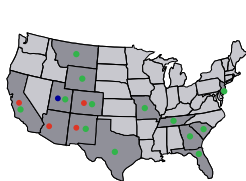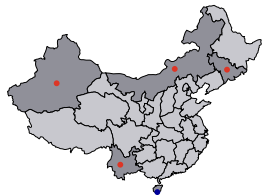

Supplement: Map S1 — Geographic distribution of isolates used in this study [file peerj-03-761-s004.pdf]
